# New-Onset and Flare Episodes of Adult-Onset Still's Disease Following COVID-19 Vaccination: A Systematic Review of Published Case Reports

**DOI:** 10.7759/cureus.100889

**Published:** 2026-01-06

**Authors:** Hairya Ajaykumar Lakhani, Omkar Prasad Baidya, Anjali Alex, Sandeep V Binorkar, Debarsi Das, Avidipta Hazra

**Affiliations:** 1 Internal Medicine, Smt. B. K. Shah Medical Institute and Research Centre, Vadodara, IND; 2 Physiology, Jagannath Gupta Institute of Medical Sciences and Hospital, Kolkata, IND; 3 General Medicine, Vadakara Cooperative Hospital, Kozhikode, IND; 4 Agadatantra (Ayurvedic Toxicology and Forensic Medicine), Government Ayurveda College, Nanded, Nanded, IND; 5 Organon of Medicine, Metropolitan Homoeopathic Medical College and Hospital, Sodepur, Kolkata, IND; 6 Homoeopathic Medicine and Surgery, Metropolitan Homoeopathic Medical College and Hospital, Kolkata, IND

**Keywords:** adult onset still disease, autoimmunity, covid-19 vaccine, immunoinflammatory response, meta analysis, post vaccination

## Abstract

This systematic review provides a descriptive synthesis of published case reports documenting new-onset or flare episodes of adult-onset Still’s disease (AOSD) temporally occurring after COVID-19 vaccination. A comprehensive search of PubMed, Scopus, Web of Science, and Google Scholar identified 13 eligible case reports published between 2020 and 2024. Because all available evidence consisted solely of individual case descriptions without comparator groups, the review followed PRISMA 2020 guidelines and employed qualitative narrative synthesis rather than meta-analysis. Across the included cases, patients consistently presented with hallmark features of AOSD, including high spiking fever, arthritis or arthralgia, markedly elevated ferritin levels, and, in several instances, the characteristic salmon-colored rash. Symptom onset typically occurred within four to fifteen days following vaccination. Although these cases demonstrate recognisable clinical patterns, the absence of denominator data, lack of population-based studies, and inherent publication bias prevent estimation of incidence or risk, and no causal relationship with vaccination can be inferred. All reported patients responded favorably to corticosteroids, with some requiring biologic therapy for disease control. These findings highlight the importance of clinician awareness when evaluating persistent febrile or inflammatory symptoms in recently vaccinated individuals, while emphasising that COVID-19 vaccination remains overwhelmingly safe. Larger registries, pharmacovigilance data, and controlled studies are needed to clarify potential risk factors and guide future revaccination decisions.

## Introduction and background

Adult-onset Still's disease (AOSD) is characterised by a unique salmon-coloured, transient rash, arthritis or arthralgia, and intermittent fever spikes [1]. Although AOSD was once considered a diagnosis of exclusion, classification criteria such as Yamaguchi and Fautrel have helped refine its definition by incorporating clinical features and inflammatory markers, including ferritin, C-reactive protein (CRP), and cytokines such as interleukin (IL)-1, IL-6, and IL-18 [2,3]. The illness primarily affects young and middle-aged adults and is not sex-based. It usually appears as chronic articular, polycyclic, or monocyclic [4]. The classic triad of daily spiking fevers, arthritis or arthralgia, and salmon-colored rash is highly suggestive of AOSD, although all three features may not always appear concurrently [1,4]. Systemic features, such as lymphadenopathy, hepatosplenomegaly, serositis, and sore throat, are also common. Laboratory findings often demonstrate leukocytosis, elevated transaminases, and profound hyperferritinemia [5]. The underlying pathogenesis involves innate immune dysregulation, including macrophage and neutrophil hyperactivation, and an overproduction of cytokines such as IL-1β, IL-6, and IL-18 [6].

Following the widespread rollout of COVID-19 vaccination, isolated reports of immune-mediated responses have emerged. Different vaccine platforms, including inactivated virus vaccines, adenoviral vector vaccines (AstraZeneca ChAdOx1 nCoV-19), and mRNA vaccines (Pfizer-BioNTech BNT162b2 and Moderna mRNA-1273), have been associated with various immune phenomena. While most individuals experience only mild, self-limiting side effects, a small subset of published case reports has described immune-mediated conditions such as autoimmune cytopenia, vasculitis, lupus-like syndromes, and AOSD temporally following vaccination [7-9].

A growing number of case reports and small case series have described temporal associations, rather than proven causal relationships, between COVID-19 vaccination and either new-onset AOSD or flares of pre-existing disease [10-14]. Several mechanistic hypotheses have been proposed, including bystander activation of antigen-presenting cells, molecular mimicry between vaccine-derived proteins and self-antigens, and heightened cytokine responses in genetically predisposed individuals [15]. Moreover, elevations in IL-18 and hyperferritinemia similar to macrophage activation syndrome (MAS) have been described in some cases [16,17]. However, these proposed mechanisms remain theoretical, and none have been empirically demonstrated to establish causation.

AOSD may have a fluctuating clinical course, with relapses occurring in 21.3% of patients; cumulative relapse rates were reported as 14.4% at six months, 21.8% at 12 months, 24.8% at 18 months, and 28.6% at 36 months [18]. Because COVID-19 vaccination was administered to billions of individuals worldwide, several AOSD flares occurring shortly after vaccination would be statistically expected to arise purely by coincidence [11]. Without baseline flare rates or denominator data on how many AOSD patients were vaccinated globally, it is impossible to determine whether the published cases exceed what would be expected by chance alone.

Despite the increasing number of published reports, there is still no consolidated descriptive review summarising the clinical presentation, laboratory abnormalities, timing of onset, and therapeutic responses of AOSD cases reported after COVID-19 vaccination. Existing reports remain geographically dispersed, heterogeneous in detail, and limited to individual observations, making it difficult for clinicians to contextualise these cases within the broader post-vaccination landscape. Because only case reports are available, the true frequency, incidence, or comparative risk of AOSD flares following vaccination cannot be determined, and temporal coincidence cannot be excluded.

Accordingly, this study aims to systematically review and descriptively synthesise all published case reports and case series of AOSD occurring after COVID-19 vaccination. In accordance with PRISMA 2020 guidelines, this review summarises the clinical features, laboratory findings, timing of symptom onset, vaccine types involved, and management approaches described in the literature [19]. The goal is not to infer causality or estimate risk, which are impossible given the absence of denominators, control groups, and population-based data, but rather to provide clinicians with a cautious, context-aware overview of the characteristics reported in the existing case-based literature.

## Review

Methods

Protocol Compliance and Guidelines

This systematic review was conducted in accordance with the PRISMA 2020 guidelines. Given that all available evidence consisted solely of individual case reports and small case series, a quantitative meta-analysis was not appropriate. Therefore, the review was designed and executed as a qualitative descriptive synthesis. All steps from database selection to screening, data extraction, and reporting adhered to PRISMA recommendations to ensure transparency, methodological rigour, and reproducibility. This review was not registered in PROSPERO because PROSPERO does not accept protocols based exclusively on case reports or case series; however, the protocol was pre-specified before the initiation of the review.

Eligibility Criteria

Eligibility was defined using a modified PICOS framework appropriate for case-based evidence. Studies were included if they reported adult patients aged 18 years or older with a confirmed diagnosis of AOSD based on established criteria such as Yamaguchi, Fautrel, or ILAR. Only studies describing new-onset AOSD or flares temporally following COVID-19 vaccination were considered. Included reports required sufficient clinical detail to confirm diagnosis, describe symptom patterns, and outline relevant laboratory findings and treatment. As case reports do not include comparison groups, no comparator arm was required. Studies were excluded if written in languages other than English, if clinical details were inadequate to confirm AOSD or a temporal relationship with vaccination, or if the report was retracted or existed only as an unverified preprint. Duplicate reports describing the same patient were identified and consolidated before final inclusion.

Information Sources

A comprehensive literature search was performed across PubMed, Scopus, Web of Science, and Google Scholar, covering the period from January 1, 2020, to December 31, 2024. This timeframe was selected to capture all case-based evidence emerging after the global introduction of COVID-19 vaccines. Additional manual screening of reference lists from relevant publications was conducted to ensure completeness. Grey literature, including institutional case summaries and academic preprints, was evaluated cautiously and included only when methodological clarity and peer-review status could be reasonably verified. Pharmacovigilance systems such as VAERS, EudraVigilance, and VigiBase were not included as primary data sources because they contain unverified spontaneous reports that do not reliably confirm AOSD diagnosis, making them unsuitable for structured extraction within a case-based systematic review.

Search Strategy

The search strategy combined Medical Subject Headings (MeSH) and free-text terms. Terms such as “Adult-Onset Still Disease,” “AOSD,” “COVID-19 vaccine,” “SARS-CoV-2 vaccination,” “mRNA vaccine,” “adenoviral vector vaccine,” “flare,” “exacerbation,” and “reactivation” were used. Boolean operators were applied to tailor search strategies for each database. The search strategy is included in Table 1.

**Table 1:** Search strategy

Search strategy	
PubMed	("Adult-Onset Still's Disease"[MeSH] OR "AOSD" OR "Still’s disease") AND ("COVID-19 Vaccines"[MeSH] OR "COVID-19 vaccine" OR "SARS-CoV-2 vaccination" OR "mRNA vaccine" OR "adenoviral vector vaccine") AND ("case report" OR "case series" OR flare OR exacerbation OR reactivation).
Scopus and Web of Science	(“adult onset still disease”) AND (“COVID-19 vaccine”) AND (case report OR flare OR exacerbation).
Google Scholar	Adult-onset Still’s disease COVID-19 vaccine case report

Selection Process

**Figure 1:**
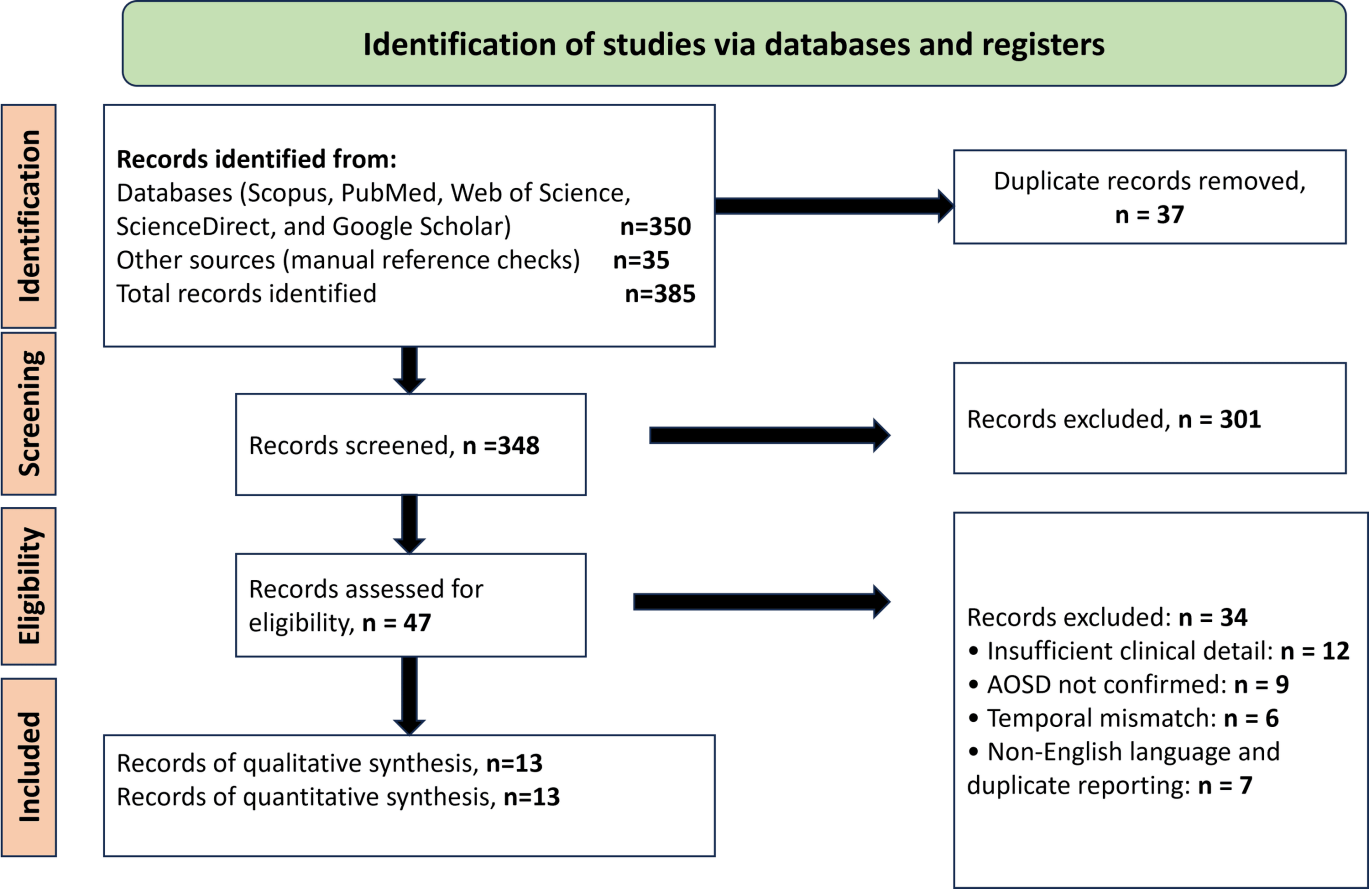
PRISMA Flow Diagram AOSD: Adult-onset Still's disease

All records identified through the search were imported into Rayyan (Rayyan Systems, Inc., Cambridge, MA) for blinded screening. Reviewers were blinded to each other’s decisions during the initial screening phase. Two reviewers independently evaluated titles and abstracts, followed by a full-text review of potentially relevant articles. Disagreements were first resolved through discussion; if consensus could not be achieved, a third reviewer adjudicated the decision. A PRISMA flow diagram summarising the screening and selection process is presented in Figure 1. This framework documents the number of articles identified, screened, assessed for eligibility, and included, along with the reasons for exclusion at each stage.

Data Extraction

Data extraction was performed using a structured Excel spreadsheet developed specifically for this review. Two reviewers independently extracted information on study identifiers, patient demographics, diagnostic criteria, clinical features of AOSD flares, relevant laboratory parameters, vaccine type and dose, interval between vaccination and symptom onset, treatment modalities, and clinical outcomes. When multiple reports described the same patient, the version containing the most complete and clinically detailed information was used to avoid duplication. Any discrepancies in extracted data were resolved by consensus or consultation with a third reviewer.

Risk of Bias Assessment

The methodological quality and risk of bias of included studies were evaluated using the Joanna Briggs Institute (JBI) Critical Appraisal Checklists designed for case reports and case series. The domains assessed included the clarity of patient selection, the diagnostic confirmation process, the adequacy of the description of the temporal relationship between vaccination and symptom onset, and the completeness of clinical and follow-up data. Because case reports inherently lack generalizability and control groups, the risk of bias across the dataset was expected to be moderate.

Data Synthesis

In accordance with PRISMA guidance for non-comparative evidence, a qualitative narrative synthesis was conducted. No statistical pooling, effect size calculation, or heterogeneity testing was undertaken because case reports cannot yield valid quantitative estimates. Instead, the synthesis focused on summarising trends across cases, including clinical manifestations, laboratory abnormalities, timing of symptom onset, implicated vaccine types, management approaches, and reported outcomes. As denominator data for vaccinated AOSD populations or global vaccination statistics were unavailable, the review does not attempt to estimate incidence, prevalence, or risk. The findings should therefore be interpreted strictly as descriptive observations of published cases. No causal associations can be inferred from these data.

Results

Study Selection

The database search initially identified 385 records from electronic databases and manual searches. After the removal of 37 duplicate entries, 348 unique articles were screened based on title and abstract. The majority of these were excluded because they did not describe AOSD flares following COVID-19 vaccination, involved non-human subjects, or lacked clinical relevance. Of the 47 full-text articles assessed, 34 were excluded for reasons including duplicate case descriptions, insufficient clinical information to confirm AOSD diagnosis, unclear temporal association with vaccination, non-English language, or lack of validated diagnostic criteria. Ultimately, 13 case reports met all inclusion criteria for the qualitative review. Because case reports represent spontaneous published observations rather than systematically collected data, the final sample reflects reporting behaviour rather than true event frequency. The study selection process is illustrated in Figure 1, which presents the PRISMA flow diagram demonstrating identification, screening, eligibility assessment, and inclusion.

Characteristics of Included Studies

The final dataset consisted solely of individual case reports describing patients who developed new-onset AOSD or experienced a flare after receiving a COVID-19 vaccine. The vaccines involved included mRNA platforms such as Pfizer-BioNTech and Moderna, as well as adenoviral vector vaccines like the AstraZeneca ChAdOx1 nCoV-19 formulation. The time between vaccination and the appearance of clinical symptoms ranged from four to fifteen days. All included reports provided adequate detail regarding symptoms, laboratory findings, and management strategies. However, the level of detail varied between reports, underscoring the heterogeneity inherent in case-based evidence. These characteristics are summarised in Table 2, which outlines the diagnostic features, vaccine types, ferritin levels, and treatments reported across cases.

**Table 2:** Summary of Included Studies Abbreviations: mRNA: Messenger ribonucleic acid, AZ: AstraZeneca, NSAIDs: Nonsteroidal anti-inflammatory drugs, IVIG: Intravenous immunoglobulin

Author (Year)	Vaccine Type	Onset (Day/s)	Fever	Arthritis	Rash	Ferritin Level (ng/mL)	Hospitalized	Treatment
Matsuda et al. [8]	mRNA (Pfizer)	7	TRUE	TRUE	TRUE	8000	TRUE	Steroids + Biologics
Leone et al. [9]	mRNA (Pfizer)	10	TRUE	TRUE	FALSE	5200	TRUE	Steroids
Kim et al. [10]	mRNA (Pfizer)	5	TRUE	TRUE	TRUE	7000	TRUE	Steroids + NSAIDs
Palassin et al. [11]	Various	12	TRUE	TRUE	TRUE	6000	TRUE	Steroids + Biologics
Sharabi et al. [12]	mRNA (Pfizer)	6	TRUE	FALSE	FALSE	4800	FALSE	NSAIDs only
Winichakoon et al. [13]	Viral Vector (AZ)	14	TRUE	TRUE	TRUE	9000	TRUE	Steroids
El Hasbani et al. [14]	mRNA (Pfizer)	8	TRUE	TRUE	FALSE	6500	TRUE	Steroids + IVIG
Liu et al. [15]	Various	9	TRUE	TRUE	TRUE	7500	TRUE	Steroids
Padiyar et al. [16]	Viral Vector (AZ)	11	TRUE	TRUE	TRUE	7200	TRUE	Steroids
Jeon et al. [17]	mRNA (Pfizer)	4	TRUE	TRUE	TRUE	8100	TRUE	Steroids + Biologics
Liozon et al. [19]	Various	15	TRUE	FALSE	FALSE	5600	TRUE	Steroids
Chua et al. [20]	mRNA (Moderna)	7	TRUE	TRUE	TRUE	7900	FALSE	NSAIDs
Muench et al. [21]	mRNA (Pfizer)	6	TRUE	TRUE	FALSE	6700	TRUE	Steroids + Biologics

Clinical Features of AOSD Flares

All 13 cases reported high spiking fever, a defining feature of AOSD. Arthritis or arthralgia occurred in eleven patients, while eight experienced the characteristic salmon-colored rash. Five patients exhibited all three hallmark features: fever, rash, and joint involvement. Additional manifestations such as lymphadenopathy or hepatosplenomegaly were reported inconsistently. Because reporting standards varied between publications, not all clinical features were uniformly documented, limiting the ability to draw comparisons across cases. The frequency of classic features across included cases is presented in Table 3.

**Table 3:** Frequency of Classic AOSD Symptoms

Clinical Feature	Number of Patients (n=13)	Proportion (%)
Fever	13	100.0
Arthritis/arthralgia	11	84.6
Salmon-colored rash	8	61.5

Laboratory Findings

All cases demonstrated substantially elevated ferritin levels, which ranged from 4,800 to 9,000 ng/mL. Other inflammatory markers, including C-reactive protein and erythrocyte sedimentation rate, were elevated in most reports, although documentation varied between studies. Additional parameters, such as interleukin-6 and liver transaminases, were noted in certain cases but were not consistently reported across all publications, limiting the potential for systematic comparison. The variability in laboratory detail further reflects the descriptive, non-standardised nature of case reports, and therefore, these findings cannot be pooled or used to estimate typical laboratory patterns.

Treatment Modalities and Outcomes

Treatment approaches varied according to symptom severity. Most patients required hospitalisation due to significant systemic inflammation, while two cases were managed in outpatient settings. Corticosteroids were the most frequently used therapy, and several patients received biologic agents, including IL-1 and IL-6 inhibitors such as anakinra and tocilizumab. One patient who developed macrophage activation syndrome was treated with intravenous immunoglobulin in addition to corticosteroids. NSAIDs alone were used in select cases with milder symptoms. Clinical outcomes were favourable across all reports, with each patient showing improvement following treatment. Outcome reporting was generally short-term, and most cases lacked extended follow-up, preventing conclusions about relapse rates or long-term disease control. These therapeutic patterns and outcomes are summarised in Table 4.

**Table 4:** Treatment Modalities and Outcomes NSAIDs: nonsteroidal anti-inflammatory drugs, IVIG: intravenous immunoglobulin

Treatment Modality	Number of Patients (n=13)	Proportion (%)
Steroids	5	38.5
Steroids + biologics	4	30.8
Steroids + NSAIDs	1	7.7
NSAIDs only	1	7.7
Steroids + IVIG	1	7.7

Risk of Bias Assessment Results

All included studies were appraised using the Joanna Briggs Institute (JBI) checklists for case reports and case series. Across the 13 reports, methodological quality was generally moderate, with consistent documentation of patient presentation, diagnostic criteria, and temporal sequence in relation to vaccination. However, variability was observed in the level of clinical detail, duration of follow-up, and reporting of investigations undertaken to exclude alternative diagnoses. No report fulfilled all JBI domains completely, which is expected for case-based evidence. The item-by-item appraisal for each study is summarised in Table 5.

**Table 5:** JBI Risk of Bias Assessment for Included Case Reports

Study	History / Presentation	Diagnostic Criteria	Temporal Sequence	Clinical Data	Follow-up	Exclusion of Alternatives	Overall Risk
Matsuda et al. [8]	Yes	Yes	Yes	Moderate	Adequate	Partial	Moderate
Leone et al. [9]	Yes	Yes	Yes	Moderate	Limited	Partial	Moderate
Kim et al. [10]	Yes	Yes	Yes	Moderate	Adequate	Partial	Moderate
Palassin et al. [11]	Yes	Yes	Yes	Moderate	Limited	Partial	Moderate
Sharabi et al. [12]	Yes	Yes	Yes	Limited	Limited	Limited	Moderate
Winichakoon et al. [13]	Yes	Yes	Yes	Moderate	Adequate	Partial	Moderate
El Hasbani et al. [14]	Yes	Yes	Yes	Moderate	Limited	Partial	Moderate
Liu et al. [15]	Yes	Yes	Yes	Limited	Limited	Limited	Moderate
Padiyar et al. [16]	Yes	Yes	Yes	Moderate	Limited	Partial	Moderate
Jeon et al. [17]	Yes	Yes	Yes	Moderate	Adequate	Partial	Moderate
Liozon et al. [19]	Yes	Yes	Yes	Limited	Limited	Limited	Moderate
Chua et al. [20]	Yes	Yes	Yes	Limited	Limited	Limited	Moderate
Muench et al. [21]	Yes	Yes	Yes	Moderate	Limited	Partial	Moderate

Discussion

This systematic review presents a consolidated descriptive synthesis of 13 published case reports documenting new-onset or flare episodes of AOSD temporally following COVID-19 vaccination. Although these cases represent geographically diverse reports, the clinical presentations described across them share strong similarities with idiopathic AOSD. Common manifestations included high spiking fever, arthritis or arthralgia, and the characteristic salmon-colored rash. Laboratory findings consistently demonstrated markedly elevated ferritin levels across all cases, reflecting significant systemic inflammation. Many individuals required systemic corticosteroids, and several ultimately received biologic therapies. The interval between vaccination and symptom onset ranged from four to fifteen days. These observations are descriptive of the reported cases and should not be interpreted as evidence of incidence, causation, or increased risk following vaccination, as denominator data for vaccinated AOSD populations are not available.

The patterns observed in these case reports are broadly consistent with prior descriptions of hyperinflammatory responses in susceptible individuals following immunologic stimulation [20]. Several case series and immunologic surveys have also noted systemic inflammatory reactions resembling autoinflammatory disease flares after various vaccine platforms [21]. Proposed mechanisms, including cytokine dysregulation and macrophage activation, parallel the pathophysiology of AOSD itself [22]. Elevations in ferritin and cytokines, particularly IL-18, have been described in both the included cases and in COVID-19-related hyperinflammatory states, supporting biological plausibility, although such observations cannot establish causation [11]. Figure 1 provides the PRISMA flow diagram summarising the selection of the included case reports that were synthesised for these interpretative observations.

Some retrospective cohort analyses referenced in the literature have reported small numbers of AOSD-like presentations following COVID-19 vaccination [23]. However, these remain rare events when considered against the backdrop of global vaccination campaigns involving billions of administered doses [19]. Importantly, the cases included in this review arise from spontaneous, published reports, which inherently reflect selective reporting of unusual or clinically severe events [24]. Milder or transient inflammatory episodes may remain unpublished. For this reason, the clustering of cases in the literature must be interpreted as a reflection of reporting behaviour rather than an indicator of frequency or risk [25]. The absence of a control group of unvaccinated AOSD patients further precludes any comparison of flare rates before and after vaccination, and any temporal association documented in these reports may represent coincidence rather than vaccine-induced immune activation [9].

Several mechanistic hypotheses have been proposed to explain how vaccination might theoretically trigger an autoinflammatory flare in predisposed individuals [17]. Molecular mimicry, in which vaccine-derived antigens share immunogenic similarities with self-peptides, could hypothetically initiate aberrant T-cell or B-cell activation [13]. Bystander activation of antigen-presenting cells and macrophages represents another theoretical pathway, particularly in individuals with genetic or immunologic susceptibility [26]. Vaccine adjuvants may also potentiate innate immune responses, although this remains speculative [10]. These hypotheses currently lack sufficient empirical validation and should be interpreted cautiously. While similarities between vaccine-associated hyperinflammation and MIS-A have been noted by some authors, these parallels serve primarily as differential diagnostic considerations rather than mechanistic proof [5].

From a clinical perspective, it is important for physicians to maintain awareness of the possibility of AOSD flares or new-onset disease when evaluating patients who develop persistent fever, rash, and musculoskeletal symptoms following recent COVID-19 vaccination [16]. Nevertheless, vaccination remains safe and essential for public health, and the extremely small number of published AOSD cases relative to the vast number of vaccinated individuals underscores the rarity of this phenomenon [27]. The documented cases, including those summarised in Tables 1-3, demonstrate favourable responses to treatment, particularly when corticosteroids and biologic agents were initiated promptly. However, the limited follow-up information in several reports prevents the determination of long-term outcomes or recurrence risk, particularly with booster doses or different vaccine platforms [22].

This review carries important limitations arising from the nature of the evidence. All included reports describe individual patients, without control groups or systematic data collection [28,29]. Consequently, incidence and risk cannot be calculated, causality cannot be inferred, and the findings reflect only the characteristics of the cases that were documented and published. Reporting bias is inherent, as unusual or severe presentations are more likely to be submitted to journals. Clinical data such as H-score, IL-18 levels, and long-term outcomes were inconsistently reported, limiting deeper comparison. Future studies using pharmacovigilance databases such as VAERS and VigiBase, as well as prospective cohort studies, may help clarify whether there is any measurable change in AOSD flare rates following COVID-19 vaccination. Additional research into genetic susceptibility, pre- and post-vaccination cytokine profiling, and predictors of hyperinflammatory reactions may further advance understanding. Development of guidance for revaccination in patients with a documented prior flare may also be valuable for clinical decision-making.

## Conclusions

This systematic review synthesises the clinical characteristics of 13 published case reports describing new-onset or flare episodes of AOSD occurring temporally after COVID-19 vaccination. The reported cases demonstrated typical AOSD features, including high fever, arthritis or arthralgia, elevated inflammatory markers, and the classic salmon-coloured rash in some instances. These findings provide a descriptive overview of published observations and should not be interpreted as evidence of incidence, increased risk, or causality. Because all available evidence derives from isolated case reports without control groups, it is not possible to determine whether vaccination influences AOSD flare rates beyond background disease variability or whether the temporal associations described are coincidental. The small number of cases and potential publication bias further limit generalisability. Nonetheless, clinicians should remain attentive when evaluating febrile or inflammatory symptoms in recently vaccinated individuals with autoinflammatory conditions. All patients recovered with timely corticosteroid therapy, with some requiring biologic agents. Future research using pharmacovigilance databases, prospective registries, and studies including unvaccinated comparator groups is necessary to clarify potential risk factors, explore underlying mechanisms, and guide recommendations for revaccination in individuals with prior AOSD flares.
